# Supplementary material for: Characteristics of hospital pediatricians and obstetricians/gynecologists working long hours in Tokushima, Japan: A cross-sectional study
Source: PLoS One. 2024 Nov 18;19(11):e0311653. doi: 10.1371/journal.pone.0311653 (PMC11573208; doi:10.1371/journal.pone.0311653)
Supplement: S1 File — (DOCX) [file pone.0311653.s001.docx]

**Q**. Please answer the following questions concerning yourself .

(As of October 1, 2021)

(1) Age　＿＿＿

(2) Sex　1. Male 2. Female 3. Other 4. None

(3) Type of work(Please check only the one that applies.)

1. Full-time physician

2. Part-time physician

3. Hospital administrator (hospital director, etc.)

(4) Main specialty (Please check only the one that applies.)

1.Pediatrics 2. Obstetrics/Gynecology 3. Others (department of ________)

**Q**. Please provide the type of medical system practiced at your primary institution.

(As of October 1, 2021)

(1) Daytime working status(Please check only the one that applies.)

1. One attending physician system (per some patients)
2. Multiple attending physician system (per some patients)
3. Working in NICU/MFICU
4. Others

(2) Nighttime or day off working status(Please check only one that applies.)

1. On-call system 2. Shift work system 3. Working in NICU/MFICU 4. Others

**Q**. Please answer the following questions about your working status at your primary institution and other institutions in the last month (September, 2021).

(1) Number of medical institutions you worked at in the last month (September, 2021)

(2) Average working hours (per week) and number of night duty/on-call shifts (per month) in the last month (September, 2021)

・Primary institution worked at:

　Average working hours per week:　＿＿＿ｈ

Number of night duty:　＿＿＿; number of day off duty:　＿＿＿; and number of on-calls:　＿＿＿; during the last month (September, 2021)

・Other institution worked at:

Average working hours per week:　＿＿＿ｈ

Number of nights duty:　＿＿＿; number of day off duty:　＿＿＿; and number of on-calls:　＿＿＿; during the last month (September, 2021)

(3)Number of days of annual paid-leave taken in the last year (2020):　＿＿＿

**Q**. Please answer the following question regarding the status of task sharing with other professionals (nurses, administrative staff, etc.) at your primary institution.

(1) Informed consent for patients (Please check only the one that applies.)

1. Always 2. Sometimes 3. Not at all

(2) Taking basic vitals and data, such as blood pressure (Please check only the one that applies.)

1. Always 2. Sometimes 3. Not at all

(3) Simple procedures to securing the intravenous line for blood sampling and intravenous infusion (Please check only the one that applies.)

1. Always 2. Sometimes 3. Not at all

(4) Inputting medical records (electronic medical records entry) (Please check only the one that applies.)

1. Always 2.Sometimes 3.Not at all

(5) Medical clerical work (preparation of medical certificates and other documents such as making patient appointments) (Please check only the one that applies.)

1. Always 2. Sometimes 3.Not at all

(6) Transporting and restocking supplies in the hospital and transporting patients to and from laboratories. (Please check only the one that applies.)

1. Always 2. Sometimes 3. Not at all

**Q**. What working style would you desire in the future? Please check all applicable options and give the specific number of hours and frequency. (The current working style is the standard. Please check all that applies.)

1. Decrease in overtime working hours per week

2. Decrease in the number of day-off duties per month

3. Decrease in the number of night duties per month

4. Decrease in the number of on-calls per month
