# Supplementary material for: Characteristics of hospital pediatricians and obstetricians/gynecologists working long hours in Tokushima, Japan: A cross-sectional study
Source: PLoS One. 2024 Nov 18;19(11):e0311653. doi: 10.1371/journal.pone.0311653 (PMC11573208; doi:10.1371/journal.pone.0311653)
Supplement: Attachment — Submitted filename: Reviewer comments_ PONE-D-24-08882.docx [file pone.0311653.s003.docx]

**Reviewer revision notes PONE-D-24-08882**

**Major comments**

- Authors undergo cross-section surveying study on pediatrics, Obstetrics, and Gynecologists in a hospital in Tokushima prefecture however the study has many limitations. The authors have highlighted most of the limitations here. However much information should be revised to accept this manuscript.
- Did the normal standardized hours for work per week differ from one institution and another? Authors should confirm this point in their analysis. did the paid leave hours differ? What is the normal or maximum paid leave days annually? It is difficult to understand the problem WITHOUT mentioning the main standardized normal values.

**Keywords**: Please remove any abbreviations from here. It will be abbreviated in the text.

**The introduction:**

**The introduction lacks much information about the main problem (Working long hours) reasons and why your study might be the initial step to solve part of the problem.**

**The authors need to mention the physician's normal standardized hours required from each institution to understand the abnormal or overtime hours.**

- **Line 103:** Japan Labor standards will be revised from FY 2024. Please mention the previous standards or highlight them so that the readers can understand at which points the update will be done.
- **Lines 83-86: Why did authors only on a study from Taiwan? More Japanese studies here will be more useful to be added here.**

**Material and methods**

- I do request to add an ethical statement because the current way is confusing and unclear. Please write a paragraph, that clarifies “**the ethics of the research****”** at the beginning of the material and methods section.
- **Line 130**: Can you add a copy template from this questionnaire in the supplementary information along with this manuscript? It is not confidential information.
- **Lines 132-134**: Did permission needed from the administrative office in the hospital to provide the information? please provide this information.
- **Line 140**: the number of participants in this study was low. It might be a big limitation in this study.
- **Line 153**: What did the authors think that sex might affect the activity of work and long-hour work in this manuscript?
- **Line 159**: Authors should add the factors affecting long hours work surveyed by the recently reported studies as <https://doi.org/10.1186/s12889-023-17531-5> here and in the discussion as well.
- **Line 188**: what is the reference of the chosen hours surveyed in this study? Please mention the reference if possible or explain why it if it a new criterion.

**Statistical analysis**

- Please mention here that each statistical test that was used was mentioned in each figure legend and mention it in each figure legend also.
- Can authors reconfirm the statistical analysis by test rather than a Chi-square test? Would be better.

**Results comments**

**Rewrite the result writing to explain the analysis done illustrating the significant elevation and decrease rather than writing Figure 1 or 2 shows….. the data explanation should be explained in a more scientific concise way. The current way just shows what is in the figure.**

- **Lines 211**: Participants work over 80 hours were very low. The titles and tasks given to these people might be important. Did the authors have any information on this?
- **Line 231, 244, and 260**: Delete the words ‘ Figure XX shows’ as the text information after it already referred to with a Figure number in brackets at the end of the paragraph.
- For figures in the text, Please follow the journal guidelines in referring to certain results; for example Figure 2-b may not be correct.

**Figures comments**

- Where are the Figure legends, I cannot see or did not see them. make a small paragraph illustrating the figure legend. Illustrate the title of the figure, figures panels, analysis done, statistical method and P-value obtained, number of participants used,…..
- In all figures, The resolution is not so good, reupload the figures with a much higher resolution.
- Remove the title from under each figure panel and remove the *P*-Value from above each Figure Panel.
- Add Figure Panel like (A) , (B) on the top left corner of each figure for better understanding. The current way is confusing and unclear.
- Show the degree of significance of each figure to let the readers understand the difference in all figures in this manuscript.

**Tables comments**

- In all tables, add the table foot paragraph illustrating the following; all analysis done in the table, number of participants, and time of analysis, …
- Use symbols in the table and write its corresponding information in the table foot.
- In Table 1, please remove the number of participants from the table title and add this information to the table foot.
- Please add the word continued on the top left corner of the page if any table exceeds more than 1 page.
- In table 1, Why ‘**None**’ is presented here. I think participants work at least in 1 institution.

**Discussion**

**The discussion need much literature concerned about factors affecting long-hour work, reasons for overtime work, and what is the recommended solution to overcome the problem of burnout.**

- **Line 295**: How did the authors think that Coronavirus pandemic can affect the pediatrics, Obs/Gyn working hours despite the difference in the specialty from those concerned with the pandemic patients? The explanation should be more clear here.
- **Line 326-327**: I think the correspondence is 2,000 to 3,000 hours not 1,000 to 2,000. Please confirm.
- **Line 316-319**: Please mention that this information is from a study done in France.
- **Line 323-324**: These hours time is per month or semester or what?
- **Line 325**: if possible, mention the overtime regulation stated by the Ministry of Labor, health in Japan for better clarification.
- **Line 343**: This study was done in France. Please find out more studies done in Japan. The salaries of physicians in Japan are not low to affect working hours. Please explain this point.
- **Line 346**: **Please state Japan's regulations for working in more than one institution in response to working hours**. This information is very important and might affect the analysis.
- **Line 367**: Please state more details of task shifting and sharing from reference 36.
- **Line 377**: information come from 2 reported papers, please check grammer here is supposed to be ‘**Studies’** not single study.

**Ethics approval and consent to participate**

Better to transfer this section to the beginning of the Material and methods section.

**Funding**

Please provide both Grant name and number here.

**Authors contributions**

Not necessary to be added regarding the journal guidelines.

**Acknowledgments**

Please add acknowledgment to the medical institutions and hospitals physicans or directors which the participants belong for permitting you to undergo this survey.
